# Taxane/gemcitabine-containing chemotherapy plus locoregional IMRT for patients with de novo metastatic nasopharyngeal carcinoma: the treatment outcomes and prognostic factors analysis

**DOI:** 10.1007/s00405-021-07192-8

**Published:** 2022-01-04

**Authors:** Chengrun Du, Mengshan Ni, Jianyun Jiang, Fangfang Kong, Ruiping Zhai, Yingchen Lv, Chaosu Hu, Hongmei Ying

**Affiliations:** 1 Department of Radiation Oncology, Fudan University Shanghai Cancer Center, Shanghai, 200032 China; 2 Department of Oncology, Shanghai Medical College, Fudan University, Shanghai, 200032 China

**Keywords:** Nasopharyngeal carcinoma, Synchronous metastasis, Baseline lymphocyte count, The subdivision of metastasis, Taxane, Gemcitabine

## Abstract

**Purpose:**

To evaluate treatment outcomes of de novo metastatic nasopharyngeal carcinoma (mNPC) patients receiving taxane/gemcitabine-containing chemotherapy followed by locoregional intensity-modulated radiotherapy (IMRT) and analyze potential prognostic factors.

**Methods:**

A total of 118 patients between March 2008 and November 2018 were retrospectively analyzed. All the patients were treated with taxane/gemcitabine-containing systemic chemotherapy followed by definitive locoregional IMRT. Potential prognostic factors including baseline absolute lymphocyte count (ALC) and the subdivision of metastasis were analyzed.

**Results:**

The median follow-up time for the whole group was 31.5 months (range 5–138 months). Of the 118 patients, 9 (7.6%) patients experienced local regional failure and 60 (50.8%) patients had progression of distant metastasis. At the time of the last follow-up, 61 (51.7%) patients were dead. The 5-year actuarial progression free survival (PFS), overall survival (OS),distant metastasis relapse free survival (DMFS) and local regional recurrence free survival (LRFS) were 34.2%, 44%, 41.1% and 82.6%, respectively. Baseline lymphocyte count ≥ 1600/μl prior to the treatment conferred better locoregional control (5y-LRFS 96% vs. 64.7%, *p* < 0.001) and distant metastasis control (5y-MFS 50.4% vs. 32.4%, *p* = 0.023). The multivariate analysis showed that high lymphocyte count was the most relevant predictor of superior PFS (HR = 0.236, *p* < 0.001) and OS (HR = 0.518, *p* = 0.04). M subdivision was found as another independent prognostic factor for OS but not for PFS.

**Conclusion:**

Taxane/gemcitabine-containing chemotherapy combined with IMRT represents an effective treatment modality for mNPC. Baseline ALC is an independent significant prognostic factor for PFS and OS.

## Introduction

Nasopharyngeal carcinoma (NPC) is prevalent in southeastern Asia. The incidence of de novo metastatic NPC (mNPC) in endemic region ranges from 6 to 8% at the time of presentation [1]. At present, there is no consensus on the therapeutic strategy for patients with mNPC because of the heterogeneity of synchronous metastatic NPC. In a recently published randomized trial [2], patients with mNPC who responded to cisplatin and 5-fluorouracil (PF regimen) were randomized to chemotherapy plus radiotherapy or chemotherapy alone. It is revealed that the addition of radiotherapy to primary tumor and nodal region significantly improved overall survival (OS). According to this randomized trial, we could select the subgroup of patients who can benefit from aggressive chemoradiotherapy based on chemotherapy sensitivity. However, it remains unclear about the efficacy of locoregional intensity-modulated radiotherapy (IMRT) combined with other chemotherapy regimens, such as taxane/gemcitabine-containing regimens. And prognostic parameters for this subgroup of patients are needed to further increase predictive accuracy.

The cancer-specific cytotoxicity immunity is believed to be important in the prevention of cancer development and progression [3, 4]. In the last decade, the novel treatments that modify the immunity, such as immune checkpoints inhibitors (ICI), have dramatically changed standard treatments in various cancers [5–7]. Lymphocytes, a marker of systemic immunity, play a critical role in the destruction of residual tumor cells and related micrometastases. The low lymphocyte count may be associated with a weak anti-tumor response and lower tumor-infiltrating lymphocytes. Especially the decrease in the subpopulation of CD8+T cell suggests that the host tends to have an inadequate immunological reaction to tumor cell [8]. Studies have shown that lymphocyte count can predict treatment outcomes in several solid malignancies [9–12]. Few literatures reported lymphocyte as an efficacy predictor of radiochemotherapy for locoregional advanced NPC [13, 14]. However, the role of lymphocyte as a prognostic factor for mNPC is still unknown.

Considering the heterogeneity of synchronous mNPC, the subdivision of metastasis was proposed. Shen et al. [15] subdivided the M1 stage into three categories: M1a, single metastasis except the liver; M1b, single lesion in the liver and/or multiple metastases in any locations except the liver; and M1c, multiple metastases in the liver. They found a significant different survival among patients with different M subdivision. The prognostic value M subdivision needed to be further confirmed in different cohort of patients, especially those receiving different treatment modality.

IMRT to primary and regional foci is routinely recommended for mNPC patients who responded to chemotherapy in our center. Taxane/gemcitabine combined with cisplatin regimens have been used as first-line regimens since 2008, which are considered more effective and convenient than PF regimen. Thus, a retrospective study was performed to evaluate treatment outcomes and potential prognostic factors including baseline ALC and the subdivision of M for mNPC patients treated in our center.

## Patients and methods

### Patients and selection criteria

**Fig. 1:**
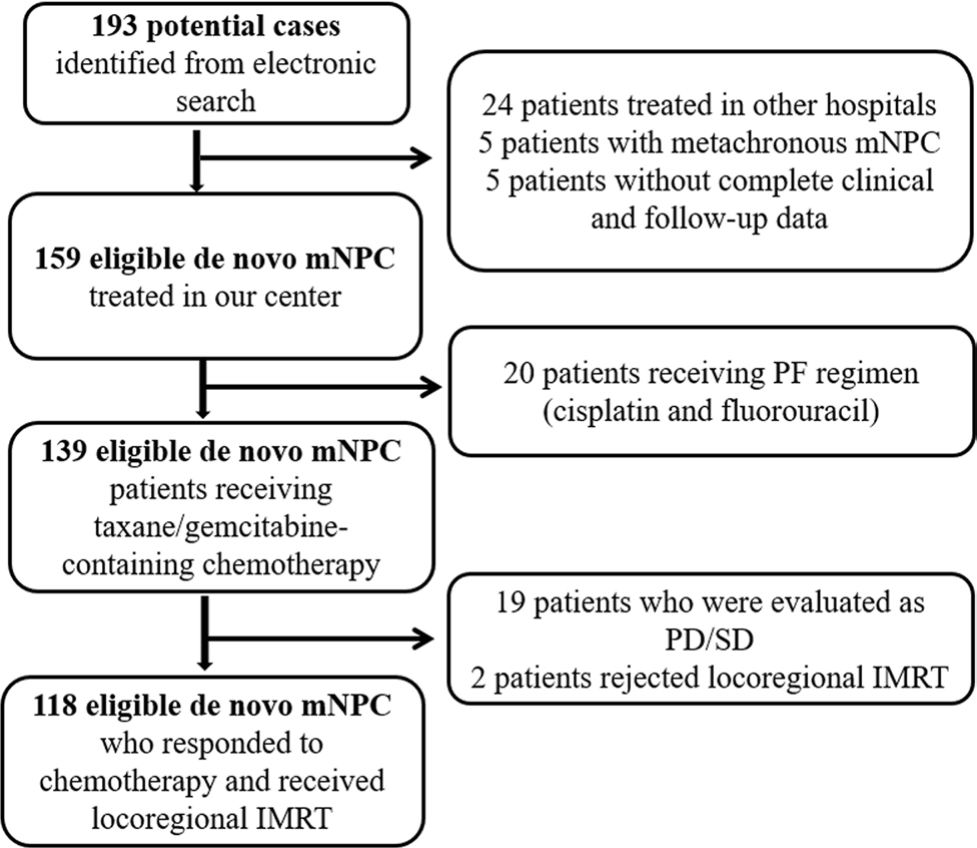
The procedure of selecting the patients

From March 2008 and November 2018, all newly diagnosed NPC patients presenting with distant metastasis at initial diagnosis who responded to chemotherapy and treated with locoregional IMRT therapy at Fudan University Shanghai Cancer Center were identified. The inclusion criteria were: (1) 18 years old and above; (2) histologically confirmed non-keratinizing and/or undifferentiated nasopharyngeal carcinoma (World Health Organization type II/III); (3) Karnofsky performance status scale ≥ 80; (4) presenting with histologically or radiologically confirmed distant metastasis at initial diagnosis.; (5) receiving chemotherapy consisting of taxane/gemcitabine combined with cisplatin, and IMRT to the head and neck region; (6) complete follow-up and clinical data. TNM stage of the patients were classified according to the eighth edition of American Joint Committee on Cancer (AJCC) staging system. The subdivision of M proposed by Shen et al. [14] was adopted in this study. Finally, 118 patents with mNPC were identified and the procedure of selecting the patients was shown in Fig. 1. Approval for the study was obtained from the institutional clinical research and ethics committee.

### Pre-treatment evaluation

Pre-treatment evaluation included complete history, physical examination, fiberoptic nasopharyngoscopy, magnetic resonance imaging (MRI) or computed tomography (CT) scan of the nasopharynx and neck regions, chest CT, ultrasonography of the abdomen, whole-body bone scan by single-photon emission CT, positron emission tomography/computed tomography (PET/CT) if necessary.

### Treatment protocol

Taxane/gemcitabine-containing chemothreapy was prescribed as follows: (1) TPF regimen: docetaxel 60 mg/m^2^/day on day 1 + cisplatin 25 mg/m^2^/day on day 1–3 + 5-fluorouracil 0.5 g/m^2^/day on day 1–3. (2) TP regimen: docetaxel 60 mg/m^2^/day on day 1 + cisplatin 25 mg/m^2^/day on day 1–3. (3) GP regimen: gemcitabine 1 g/m^2^/day on day 1,8 + cisplatin 25 mg/m^2^/day on day 1–3. All regimens were recycled intravenously every 3 weeks. Ninety-one (77.1%) patients were treated with TPF/TP regimens and 27 (22.9%) patients received GP regimen. The median number of cycles of chemotherapy for all patients was six (range 4–6).

Patients who responded to chemotherapy received IMRT to the head and neck region after the completion of 4–6 cycles chemotherapy. The prescribed dose was 60–70.4 Gy to the planning target volume (PTV) of the gross tumor volume of nasopharynx (GTVnx) in 30–35 fractions (2–2.2 Gy per fraction), 60–70 Gy to the PTV of the gross tumor volume of metastatic neck lymph nodes (GTVnd), 60 Gy to the PTV of the high-risk region (clinical target volume [CTV1]) and 54 Gy to the PTV of the low-risk region (CTV2). The median dose was 66 Gy (range, 59.4–75.4 Gy). Eighty-nine (75.4%) patients received the radical radiotherapy (≥ 66 Gy) and 29 (24.6%) patients underwent a dose < 66 Gy. Seven (5.4%) patients received concurrent chemotherapy using cisplatin 80 mg/m^2^ given intravenously every 3 weeks for two cycles.

Totally, 39 (33.1%) patients received local therapy to metastatic sites. Palliative radiotherapy to the bone lesions (30–40 Gy/10–20 fractions) was given in 33 patients for the purpose of pain alleviation. Four (3.4%) patients received surgery for vertebral metastases. Interventional or radiofrequency ablation surgery were used to treat liver lesions in 6(5%) patients. For lung metastatic lesions, two patients received SBRT and one patient underwent surgery.

### Lymphocyte count examination

Blood tests were routinely performed before chemotherapy and lymphocyte count data were available at our Hospital’s laboratory database. Lymphocyte count within 1 week before chemotherapy was collected for analysis in this study. The cut-off value of 1600/μl demonstrating maximum sensitivity and specificity (Youden Index) was determined based on receiver operating characteristic curves (ROC). The patients were classified into high absolute lymphocyte count (ALC) group (≥ 1600/μl) and low ALC group (< 1600/μl) based on the cut-off value for further analysis.

### Outcomes and follow-up

Our primary study endpoint was progression-free survival (PFS), which was defined as time from the first day of treatment to locoregional or distant metastasis progression or death from any cause or the recent follow-up for censor. The secondary endpoints included overall survival (OS), which was calculated from the first day of treatment until the day of death from any cause or the recent follow-up for censor, distant metastasis-relapse free survival (DMFS), defined as the duration from the first day of treatment to the date of distant relapse or the recent follow-up for censor, and locoregional-recurrence free survival (LRFS), defined as the duration from the first day of treatment to the date of locoregional-recurrence or the recent follow-up for censor.

After completion of treatment, all patients were followed up every 3 months during the first 2 years and every 6 months in the third to fifth year and annually thereafter. Patients underwent physical examination, including indirect nasopharyngoscopy and palpation of neck lymph nodes. In addition, MRI or CT of nasopharynx, chest CT scan, abdominal ultrasonography and radiologic imaging (CT, MRI or ultrasonography) of the distant metastasis were carried out in the third month and every 6 months afterward. Treatment-related toxicities were assessed according to Common Terminology Criteria for Adverse Events (version 4.0).

### Statistical analysis

The interactions of clinical pathological factors and pretreatment ALC were analyzed using *χ*^2^-test (or Fischer’s exact test, if indicated). Survival functions were estimated by the Kaplan–Meier method. Potential prognostic factors including patients’ clinical characteristics (sex, age), tumor features (T classification, N classification, M subdivision, number of metastatic sites), number of chemotherapy cycles, dose of radiotherapy and baseline lymphocyte counts (high lymphocyte counts vs. low lymphocytes counts) were evaluated using log-rank test. Univariate and multivariate analysis were performed using the Cox proportional hazards model. Hazard ratios (HR) and 95% CI were estimated. The multivariate analyses were undertaken with forward stepwise procedures for identifying variables correlated with OS and PFS. Any result with two-tailed *p* value < 0.05 was considered statistically significant. Data analysis was performed using the SPSS version 24.0 (IBM, Armonk, NY, USA).

## Results

### Patient characteristics and overall clinical outcomes

**Fig. 2:**
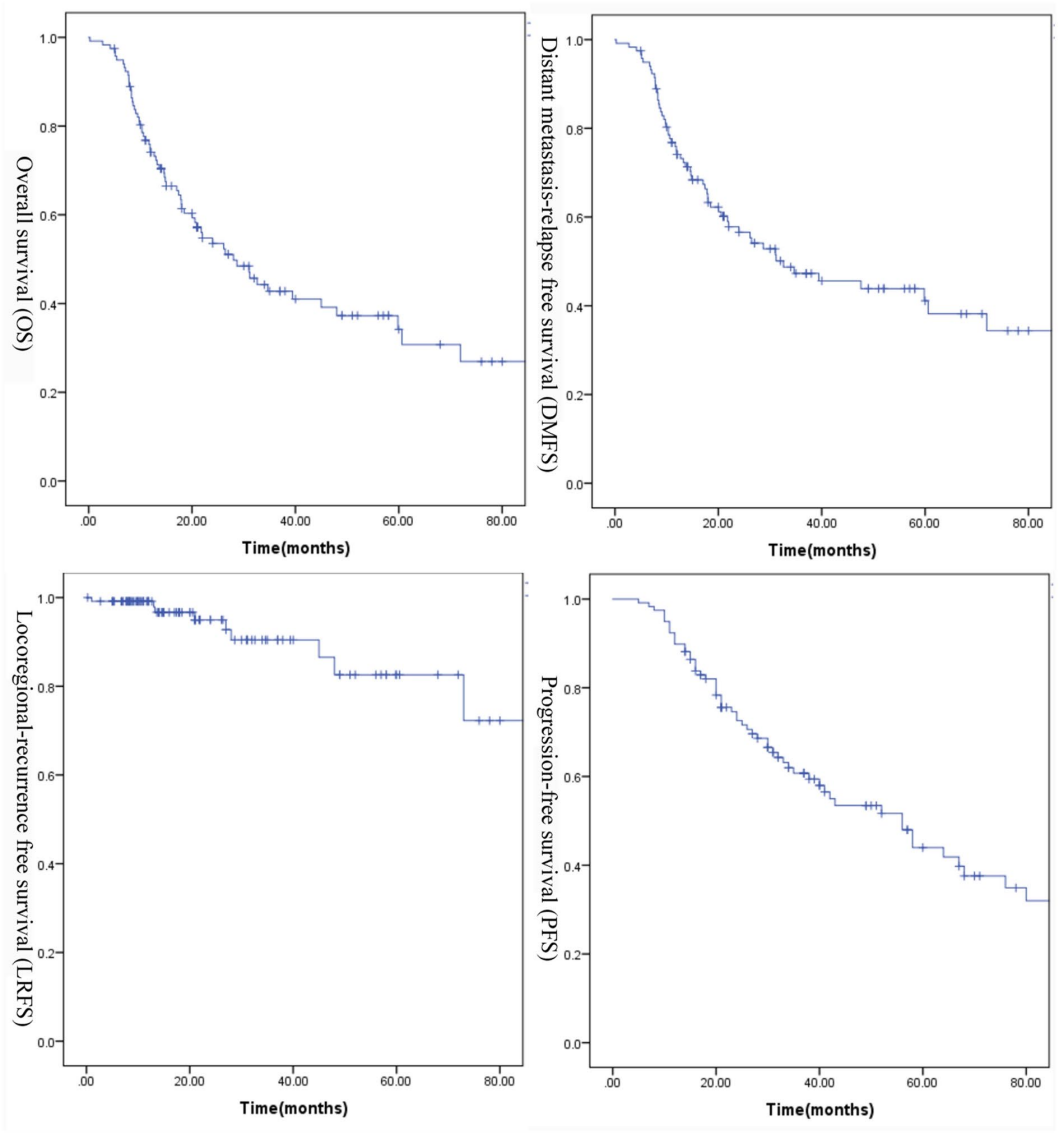
Kaplan–Meier estimates of survival curves for entire population

The characteristics of the 118 patients are presented in Table 1. The median follow-up time for the whole group was 31.5 months (range 5–138 months). Of the 118 patients, 9 (7.6%) patients experienced local regional failure, and 60 (50.8%) patients had progression of distant metastasis. At the time of the last follow-up, 61 (51.7%) patients were dead. The 5-year actuarial progression free survival (PFS), overall survival (OS),distant metastasis relapse free survival (DMFS) and local regional recurrence free survival (LRFS) were 34.2%, 44%, 41.1% and 82.6%, respectively (Fig. 2).

**Table 1:** Characteristics of all the patients

Characteristic	Number of patients	Percentage (%)
Age(years)		
≤ 50	52	44.1
> 50	66	55.9
Sex		
Male	90	76.3
Female	28	23.7
Tumor classification^a^		
T1	19	16.1
T2	20	16.9
T3	53	44.9
T4	26	22
Nodal classification^a^		
N0	3	2.5
N1	16	13.6
N2	42	35.6
N3	57	48.3
Sites of metastasis		
Bone	94	79.7
Single/ multiple	34/60	28.9/42.4
Lung	22	18.6
Single/ multiple	7/15	5.9/12.7
Liver	16	13.6
Single/ multiple	7/9	5.9/7.6
M1subdivision^b^		
M1a	41	34.7
M1b	70	59.3
M1c	7	5.9
Number of metastatic lesions		
≤ 5	33	27.9
> 5	85	72.1
Radiation dose of the nasopharynx and neck (Gy)		
< 66	29	24.6
≥ 66	89	75.4
Local treatment of metastatic disease		
Yes	39	33.1
No	79	66.9
Concurrent chemotherapy		
Yes	6	5.1
No	112	94.9

^a^Tumor-node-metastasis staging system proposed by the American Joint Committee on Cancer (8th edition)

^b^M1 stage subdivision: M1a, single lesion confined to an isolated organ or location except the liver; M1b, single lesion in the liver and/or multiple lesions in any organs or locations except the liver; and M1c, multiple lesions in the liver

### Prognostic factors associated with PFS and OS

**Fig. 3:**
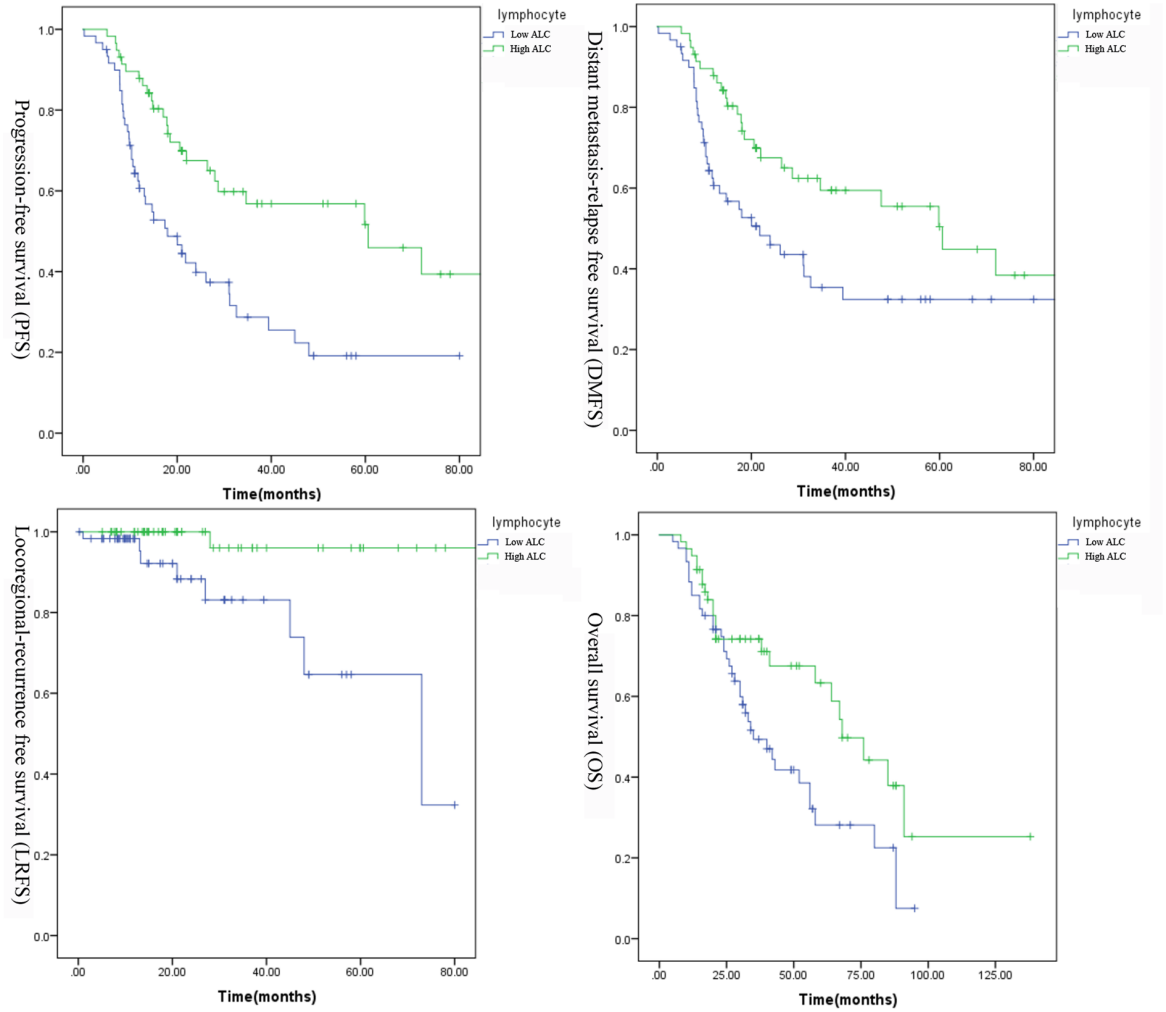
Kaplan–Meier estimates of survival curves according to baseline lymphocyte count

The results of univariate and multivariate analyses of potential prognostic factors associated with PFS and OS are shown in Table 2. Patients with high ALC had a significantly better 5y-PFS than that of patients with low ALC (5y-PFS 51.6% vs. 19.1%, *p* < 0.001). Although the favorable factors associated with PFS included bone metastasis, lung metastasis, local treatment for metastatic lesions, without concurrent chemotherapy and chemotherapy cycles in univariate analysis, the multivariate analysis showed that high lymphocyte count and without local treatment to metastatic lesions were the relevant independent predictors of superior PFS (HR = 0.236, *p* < 0.001). Overall survival of patients with high ALC was significant higher than that of those with low ALC (5y-OS 63.3% vs. 28.1%, *p* = 0.016). High baseline ALC remained a favorable prognostic factor in multivariate analysis (HR = 0.518, *p* = 0.04). Baseline lymphocyte count ≥ 1600/μl prior to the treatment also predicted better locoregional control (5y-LRFS 96% vs. 64.7%, *p* < 0.001) and distant metastasis control (5y-MFS 50.4% vs. 32.4%, *p* = 0.023) (Fig. 3).

**Table 2:** Univariate and multivariate analyses of factors associated with progression-free survival and overall survival

Characteristic	Progression-free survival			Overall survival			
	Univariate	Multivariate		Univariate		Multivariate	
	*p* value	HR	*p* value	*p* value		HR	*p* value
Age(years)	0.2			0.839			
≤ 50							
> 50							
Sex	0.533			0.476			
Male							
Female							
Tumor classification^a^	0.426			0.435			
T1							
T2							
T3							
T4							
Nodal classification^a^	0.334			0.371			
N0							
N1							
N2							
N3							
Bone metastasis	0.025		0.221	0.797			
Yes		1.784					
No		1					
Lung metastasis	0.04		0.402	0.082			0.108
Yes		0.673				0.823	
No		1				1	
Liver metastasis	0.942			0.417			
Yes							
No							
M1subdivision^b^	0.399			0.003			< 0.001
M1a						1	
M1b						4.107	< 0.001
M1c						3.083	0.057
Number of metastatic lesions	0.158			0.052			0.704
≤ 5						1	
> 5						0.892	
Radiation dose of the nasopharynx and neck(Gy)	0.189			0.109			
< 66							
≥ 66							
Local treatment of metastatic disease	0.001		0.001	0.428			
Yes		2.339	0.001				
No		1					
Chemotherapy regimens	0.724			0.884			
Docetaxel							
Gemcitabine							
Concurrent chemotherapy	0.003		0.13	0.038			0.173
Yes		2.088			2.055		
No		1			1		
Lymphocyte	< 0.001		< 0.001	0.016			0.045
< 1.6		1			1		
≥ 1.6		0.34			0.595		

^a^Tumor-node-metastasis staging system proposed by the American Joint Committee on Cancer (8th edition)

^b^M1 stage subdivision: M1a, single lesion confined to an isolated organ or location except the liver; M1b, single lesion in the liver and/or multiple lesions in any organs or locations except the liver; and M1c, multiple lesions in the liver

M subdivision was found as another independent prognostic factor for OS but not for PFS (Table 2). Patients with M1a (single lesion confined to an isolated organ or location except the liver) experienced a superior OS compared with those with M1b and M1c.

### Toxicities

Toxicity analysis of all the patients was shown in Table 3. Leukopenia and neutropenia were the main adverse effect. Thirty-eight and 44 patients experienced grade III–IV leukocytopenia and neutropenia, respectively. Non-hematological adverse events included alopecia, nausea, vomiting and diarrhea, most of which were grade I-II. The incidence of grade 3 radiotherapy-related mucositis was 27%, and no grade 4 acute mucositis was observed. Twenty-six patients had suffered grade 2 weight loss. No grade 3 or grade 4 hematological toxicity occurred during RT.

**Table 3:** Toxicities of all the patients

Toxicity	Grade 0	Grade 1	Grade 2	Grade3	Grade 4
	*n* (%)	*n* (%)	*n* (%)	*n* (%)	*n* (%)
Leucopenia	21 (18)	21 (18)	38 (32)	34 (29)	4 (3)
Neutropenia	28 (24)	15 (13)	32 (27)	21 (18)	22 (19)
Anemia	32 (27)	42 (36)	30 (25)	12 (10)	2 (2)
Thrombocytopenia	61 (52)	28 (24)	16 (14)	8 (7)	5 (4)
Hepatotoxic event	90 (76)	18 (15)	7 (6)	3 (3)	0
Nephrotoxic event	97 (82)	18 (15)	3 (3)	0	0
Alopecia	59 (50)	54 (45)	5 (4)	0	0
Diarrhea	110 (93)	6 (5)	2 (2)	0	0
Nausea	0 (0)	63 (53)	40 (34)	15 (12)	0
Vomiting	51 (43)	30 (25)	37 (31)	1 (1)	0
Mucositis	0	21 (18)	66 (56)	31 (27)	0
Skin reaction	0	102 (86)	16 (14)	0	0
Weight loss	39 (33)	53 (45)	26 (22)	0	0

## Discussion

NPC patients with synchronous distant metastasis face poor prognosis, unlike those without metastasis with up to 90% of 5-year overall survival. Although no randomized evidence has showed an improvement in survival using chemotherapy compared with best supportive care alone, Chen et al. found a 53.2% reduction in the risk of death in patients undergoing chemotherapy compared to patients undergoing supportive treatment in respective study [16]. Standard treatment comprises chemotherapy with platinum doublets of drugs such as gemcitabine, paclitaxel, and 5-FU together with cisplatin/carboplatin. For treatment-naive patients who receive platinum-based chemotherapy, response rates as high as 80% and a median survival of 12–18 months may be achieved [17]. Radiotherapy is usually employed for palliative purpose for metastatic cancer, such as alleviation of pain and relieving symptoms. However, some studies have demonstrated that intensive local therapy could prolong OS in treatment-naive metastatic cancers, reflecting that certain subgroup of patients with newly mNPC may obtain long-term survival after aggressive treatment [18, 19]. Recently, Chen et al. [2] randomized 126 mNPC who responded to PF regimen to chemotherapy plus locoregional radiotherapy or chemotherapy alone in a phase III randomized trial. It is revealed that the addition of radiotherapy improved survival outcomes, with 2-y OS of 76.4% and PFS of 35%. It seems that the sensitivity to chemotherapy is critical to select the patients who could benefit from locoregional radiotherapy. However, it remains unknown about the efficacy of locoregionl intensity-modulated radiotherapy (IMRT) combined with other chemotherapy regimens, such as taxane/gemcitabine-containing regimens.

Gemcitabine plus cisplatin (GP) is considered as the standard first-line treatment for recurrent and metastatic NPC, evidenced by the phase III trial demonstrating that GP regimen prolonged median progression-free survival from 5.6 months to 7 months compared with classical PF regimen [20]. Taxane combined with cisplatin, such as TPF/TP regimen, is also proved to be effective in locoregionally advanced NPC [21, 22]. In our center, taxane/gemcitabine combining with cisplatin regimens have been routinely recommended for mNPC since 2008. This study analyzed the treatment results of 118 who responded to GP or TPF/TP regimen and then treated with locoregional IMRT. The data presented in this study demonstrated that taxane/gemcitabine plus cisplatin combined with IMRT represented an effective treatment for mNPC, with OS and PFS being 44% and 34.2%, respectively. 2-year PFS of 53.6% in our study is better than that reported by the randomized trial. The superiority in PFS may be contributed to the novel drug taxane and gemcitabine. Thus, taxane/gemcitabine plus cisplatin combined with IMRT has the potential to improve survival outcomes in newly mNPC with mild toxicity and is worthy of further investigation in prospective studies.

In the present study, pretreatment lymphocyte count was found to be an independent prognostic factor for both PFS and OS in the univariate and multivariate analysis. Among patients with high pretreatment ALC, only one patient (1/58, 1.7%) experienced locoregional relapse, while eight patients (8/60, 13.3%) in low ALC group had locoregional failure. Patients with high lymphocyte count also experienced lower rate of distant progression. Lymphocytes, an essential component of host immunity, play a critical role in the destruction of residual tumor cells and micrometastases. The effect of chemotherapy and radiotherapy is found to be associated with the function of lymphocytes. Its prognostic value has been reported in the locoregional advanced NPC without distant metastasis. Liu et al. [13] collected serial lymphocyte counts and survival data from 413 NPC patients, and demonstrated that low mini-ALC during treatment predicted a worse PFS and OS. To our knowledge, this study is the first study to investigate the role of lymphocyte as prognostic factor in NPC with synchronous metastasis. We did not analyze the role of serial lymphocyte counts during or after treatment since the lymphocyte count are vulnerable to chemoradiation and can be increased by some supportive treatments, such as dexamethasone and granulocyte–macrophage colony-stimulating factor (GM-CSF). The confounder factors would reduce the capacity of ALC to reflecting status of immunity and impede us to investigate prognostic value of ALC. Baseline ALC can be more feasibly and consistently obtained and thus the predicting value based on it would be more likely to be repeated and generalized.

PD-1 inhibitor therapy has revolutionized the treatment of cancers, including NPC [6]. In the management of recurrent or metastatic NPC, pembrolizumab and nivolumab, are now used as standard second line agents [23, 24]. The combination of camrelizumab with cisplatin and gemcitabine used as first-line therapy in 23 patients with recurrent or metastatic demonstrated an objective response of 91% [25]. Trials investigating PD-1 inhibitor therapy in the de novo mNPC are ongoing. However, the predictive biomarkers of PD1 inhibitor therapy other than PD-L1 expression, which is of limited utility in NPC, are much in need. Since the mechanism of anti-PD-1 antibodies is thought to be dependent on the activity of functional T lymphocytes, it is rational to hypothesize that the efficacy of anti-PD1 antibodies would be compromised in patients with low lymphocyte count. Ho et al. [26] treated 34 recurrent/metastatic head and neck patients with either nivolumab or pembrolizumab alone and found that lower ALC was significantly associated with lack of clinical benefit. Therefore, baseline ALC, not only predicting the outcomes of chemoradiation, but also relevant to efficacy of PD-1 inhibitor therapy, should be considered in future prospective studies on PD-1 inhibitor therapy for mNPC.

mNPC patients is a heterogeneous group of patients exhibiting a wide range of survival outcomes. M1 classification cannot be used to accurately predict survival outcomes among mNPC patients. The subdivision of M1 stage based on involved organs and the number of lesions has been proposed. Zou et al. [19] subdivided the M1 stage into three categories: M1a, oligo metastasis without liver involvement; M1b, multiple metastases without liver involvement; and M1c, liver involvement irrespective of metastatic lesions. They found that the M1a and M1b classification of NPC may benefit from aggressive and potentially curative treatment, while chemoradiotherapy did not benefit patients in M1c. Shen et al. [15] classified M1 stage into three groups: M1a, single lesion confined to an isolated organ or location except the liver; M1b, single lesion in the liver and/or multiple lesions in any organs or locations except the liver; and M1c, multiple lesions in the liver. The subdivision by Shen et al. [14], adopted in this study, was found be an independent factor for OS, but not for PFS. Patients with M1b had significantly worse OS than patients with M1a (HR = 4.107, *p* < 0.001), and for patients with M1c accounting for seven patients, there is a trend of inferior survival compared with M1a. It is demonstrated that the subdivision of M1 is still relevant to prognosis even in chemotherapy-sensitive patients who can benefit from radical locoregional RT and should be considered in tailoring the treatment for mNPC.

Other treatment-related factors, such as the cycle of chemotherapy [18, 19] and radiation dose [27, 28] were found to be related to treatment outcomes in previous studies. However, none of these was found to be an independent prognostic factor in our study. This inconsistency could be partially explained by that the previous studies have a large heterogeneity in the treatment modality and patients recruited. In our study, patients received relatively uniform treatment. All the patients received 4–6 cycles of chemotherapy. Equal to or more than 60 Gy of total dose was given to all the patients and 89(75.4%) patients received 66–70.4 Gy. Local treatment of metastatic lesions was associated with worse survival outcomes in our study, which can be explained that local treatments of metastatic lesions were mainly employed to for palliative purpose to alleviate local symptoms in patients with progressive diseases.

This study was subject to several limitations. Firstly, due to the respective nature and limited sample size, all analyses are subject to the influences of selection bias and potential imbalances in unquantified variables. Secondly, our data did not analyze the role of lymphocyte subpopulations, as lymphocyte having both pro- and anti-cancer function [29, 30]. Furthermore, circulating EBV DNA load was unknown in the majority of patients. The predicting modal based on lymphocytes and circulating EBV DNA may be more accurate to individualized treatments for mNPC, which is our future research direction. Additionally, it cannot be determined from our data whether lymphopenia is a truly predictive biomarker of aggressive chemoradiotherapy, or simply a global prognostic biomarker. Finally, our data were exclusively obtained from one center; therefore, these results must be validated by other datasets.

In conclusion, taxane/gemcitabine plus cisplatin combined with IMRT represents an effective treatment modality for synchronous mNPC. Baseline ALC is an independent prognostic factor for PFS and OS. The subdivision of M1 is still correlated with OS in the chemo-sensitive patients receiving locoregional IMRT. Further prospective studies are warranted to investigate the treatment alternative and potential prognostic factors found in this study.

## Data Availability

All the data and material are available.
